# Using movies to teach professionalism to medical students

**DOI:** 10.1186/1472-6920-11-60

**Published:** 2011-08-23

**Authors:** Zalika Klemenc-Ketis, Janko Kersnik

**Affiliations:** 1 Department of Family Medicine, Medical School, University of Maribor, Slomskov trg 15, 2000 Maribor, Slovenia; 2 Department of Family Medicine, Medical School, University of Ljubljana, Poljanski nasip 58, 1000 Ljubljana, Slovenia

## Abstract

**Background:**

Professionalism topics are usually not covered as a separate lesson within formal curriculum, but in subtler and less officially recognized educational activities, which makes them difficult to teach and assess. Interactive methods (e.g. movies) could be efficient teaching methods but are rarely studied. The aims of this study were: 1) to test the relevance and usefulness of movies in teaching professionalism to fourth year medical students and, 2) to assess the impact of this teaching method on students' attitudes towards some professionalism topics.

**Method:**

This was an education study with qualitative data analysis in a group of eleven fourth year medical students from the Medical School of University Maribor who attended an elective four month course on professionalism. There were 8 (66.7%) female students in the group. The mean age of the students was 21.9 ± 0.9 years. The authors used students' written reports and oral presentations as the basis for qualitative analysis using thematic codes.

**Results:**

Students recognised the following dimensions in the movie: communication, empathy, doctors' personal interests and palliative care. It also made them think about their attitudes towards life, death and dying.

**Conclusions:**

The controlled environment of movies successfully enables students to explore their values, beliefs, and attitudes towards features of professionalism without feeling that their personal integrity had been threatened. Interactive teaching methods could become an indispensible aid in teaching professionalism to new generations.

## Background

Professionalism can be defined as a collection of attitudes, values, behaviours and relationships that act as the foundation of the health profession's contract with society [1]. It is an essential ability to be instilled in medical students, alongside biomedical knowledge and clinical skills [2]. The process of professionalism attainment is very long and affected by many factors among which the education process is regarded to be the crucial one [3].

Professionalism topics are often a part of a so called "hidden curriculum", which means that its determinants do not operate within the formal curriculum as a separate lesson, but in a more subtle and less officially recognized educational activity, which makes them difficult to teach but nevertheless should be taught [4]. Interactive teaching methods using art as a teaching tool are very efficient [5,6]. Movies, for example, present developed scenarios and are a form of controlled environment, which enables reproducible, focused and independent student learning. Through art, students are able to understand patients in their whole context [7]. The use of movie clips or whole movies to help educate learners about bio-psycho-social-spiritual aspects of health care - cinemeducation [8], has been widely used in medical education [7,9-13]. It has been shown that students received this teaching method well [7,8,10,11] and that it was particularly good in the teaching of patients' care, communication skills, breaking bad news, ethical issues and family dynamics [6,8,10-12].

Interactive methods could be efficient teaching methods but are rarely studied. So, the authors decided to perform a study about the relevance of including movies in the curriculum. They chose the movie Wit. It deals with the very personal story of a professor of English literature dying of metastatic ovarian cancer and describes her experiences with medical care from first encounter with the diagnosis to her death. It deals with many professionalism virtues, such as altruism, humanism, confidentiality and patient autonomy [14,15]. The aims of this study were; 1) to test the relevance and usefulness of movies in teaching professionalism to fourth year medical students and, 2) to assess the impact of this teaching method on students' attitudes towards some professionalism topics: positive and negative patients' communication elements, empathy and the effect of the movie on students' personal attitudes towards death and dying patients.

## Methods

### Study design

This was an education study with qualitative data analysis.

### Study sample and setting

In the academic year 2010-2011, fourth year medical students of the Medical School of University Maribor, Slovenia, could choose among several elective courses, including one from the field of family medicine. The topic was professionalism in medicine. All 11 students that chose this topic also participated in the project. There were 8 (66.7%) female students in the group. The mean age ± SD of the students was 21.9 ± 0.9 years.

### Course description

This elective course lasted for 4 months (Table 1). The following teaching methods were used: lectures, group work with discussion and individual work. For the group work, students were divided into three groups. Teachers used the following methods of assessment: marking an essay and an oral presentation of the topic.

**Table 1:** Time table and content of the course

Time	Content	Method	Home assignment
Day 1	Introduction to professionalism and information about the course	Lecture	Watching a movie

Day 8	Doctor/patient communication, ethical issues and the role of doctor and nurse in palliative care	Group work Plenary discussion	Watching a movie with a special emphasis on the topic chosen

Day 30	Professionalism and humanism in medicine	Lecture Group work Plenary discussion	Writing an essay on a chosen topic

Day 60	Doctor/patient communication, ethical issues and the role of doctor and nurse in palliative care	Plenary discussion	Writing an essay on a chosen topic

Day 90	Palliative care	Lecture Group work Plenary discussion	Preparing oral presentation on a chosen topic

Day 120	Doctor/patient communication, ethical issues and the role of doctor and nurse in palliative care	Plenary presentation Discussion	

Topics for lectures were chosen by a teacher (ZKK) and topics for three groups (doctor/patient communication, ethical issues and the role of doctor and nurse in palliative care) were chosen during an introductory session by the students themselves. Two sessions with in depth discussions about the professional issues covered in the film followed and at the end of the course, students had to write an essay and give an oral presentation on a chosen topic.

### Instrument and analysis

In the essays, students had to describe and discuss their observations about the movie and the impact of the movie on their attitudes towards the three topics they chose to be important in the movie at the beginning of the course. The essays were presented as free text, but the teacher instructed students to incorporate in the text the answers to the following questions: 1) which positive behavioural, communication and consultation elements of health professionals they found in this film according to the chosen topic, 2) which negative behavioural, communication and consultation elements of health professionals they found in this film according to the chosen topic, 3) which such topics they recognised in the movie besides the general ones, 4) what would they do in similar situations as seen in the movie, 5) how did the movie affect their feelings, beliefs, values, and 6) how do they think the movie will influence their future professional and personal life.

Essays and oral presentations were evaluated by one teacher (ZKK) using grounded theory based coding method (open coding) [16]. She read the essays and defined discrete essay segments. When analysing oral presentations, she recorded the narrative statements. Each segment of an essay and each narrative statement were then coded with a short phrase, which best described the theme. Sections of text and narrative statements could be assigned multiple codes. After the coding, the teacher summarized the prevalence of codes, analysed the similarities and differences and came up with several final themes [17].

### Outcome measures

Outcome measures were individual attendance in general, and film specific professional themes discussions during the course, and identification, elaboration and reflection of the themes in written assignments and presentations. The authors regarded mentioning themes, such as facing death in a patient, breaking bad news to the patient, communication skills, consultation skills and human behaviour in health care professionals, as essential themes for this educational programme. They were identified in written assignments and presentations.

## Results

Students recognised the following dimensions in the movie: communication, empathy, doctors' personal interests and palliative care. They also reported that this movie made them think about their own life and death, and helped them to understand all phases of a dying patient.

### Communication

Students recognised that communication was an essential part of medicine. *"A doctor-patient communication seems as the most important element of healing and of health itself. Namely, with good communication, we can heal or relieve many patients' problems or worries."*

Students stressed the importance of non-verbal communication as an essential part of good communication. They noticed wrong ways of doctor-patient non-verbal communication, presented in this movie: lack of eye contact, lack of physical contact, lack of noticing patient's non-verbal signs and wrong reaction to patient's non-verbal signs. *"Doctor gave the impression that he had no interest to get closer to patient and thought that an occasional smile will calm every patient's worries." *On the other hand, students noticed right ways of nurse-patient non-verbal communication, presented in this movie: good eye contact, physical contact, noticing and responding to patient's non-verbal signs. *"Among non-verbal elements, nurse-patient relationship was based on small things, i.e. nurse gave an ice-stick to the patient. With these small things, nurse made it clear that patient can always rely on her."*

Another part of good communication, as observed by students, was good listening abilities of the doctors, i.e. active listening and following patients' clues. *"The doctor listened but did not hear her. He seemed to have asked all those questions just because he had to do so, not because he really wanted to find out what she felt." "The doctor did not allow the patient to express her fears and expectations."*

Accessibility and openness were also recognised as important parts of doctors' behaviour during communication. Doctors should not have used too much jargon when communicating with the patient in this movie. This made them inaccessible.

"Such patients do not need statistical data and a basket, filled with medical terms which they do not understand; they need emotional support, time, good explanation and understanding."

### Empathy

Students recognised that a doctor-patient relationship should be based on empathy, which is also the key issue for good management of a dying patient. *"The whole point is in listening and empathy." *They described a doctor-patient relationship, presented in this movie, as cold, emotionless and too rational. *"The doctor regarded the patient as an object. In her, he saw just another case of illness. He acted totally unprofessional." *On the other hand, the students easily recognised the empathy, provided in this movie by a nurse. *"Nurse regarded this patient as a subject. She saw her as a person who is breathing, thinking, hearing, seeing, talking, wanting, suffering and seeking support from other people."*

### Doctors' personal interests

Students reported that when dealing with patients, doctors should forget about their personal wishes and goals. They saw doctors in the movie as overly scientific and research-oriented and ignorant about real patients' needs. *"Doctor forced her to agree to the proposed treatment and then he even insisted on the highest dosages of the drug - even though it was clear that the drug is doing more harm than benefit to the patient."*

Students recognised that doctors should not regard the death of their patients as their personal failures. *"Doctors in the movie regarded the patient's illness as their own failure so they were embarrassed when being around her."*

### Palliative care

Students recognised that doctors should also provide palliative care and that such care was also a kind of treatment, where *"treating symptoms is as important as pain management and providing psychological support." *Students accepted that the doctors' role is also to enable patients to prepare for their own death. *"Management of a dying patient is as important as management of a patient with any other disease."*

Students stressed that when dealing with a dying patient, it is very important that doctors act honestly and respect patients' autonomy. *"Doctor and patient should agree about the course of the treatment and hence achieve a balance between honesty and autonomy." *Also, they thought that doctors should plan palliative care in agreement with the patient - especially the question of resuscitation. *"During constant worsening of patient's clinical state, nobody (except nurse) had talked about death and a possibility of non-resuscitation."*

Students regarded the doctor's management of their patient in this movie as one-sided and purely bio-medically founded. On the other hand, students regarded the nurse's management of the patient in this movie as comprehensive and holistic. *"For nurse*, *living meant to live as long as life has a meaning. For doctors, life was determined only by patient's vital functions."*

### Students' attitudes towards their own life, death and dying

Students reported that this movie made them think about human death and dying in general. They came to the conclusion that all patients react the same way when faced with their own death. They also expressed that this film helped them to understand all phases of a dying patient. *"Future doctors should learn how to identify with the patients, how to show them a proper degree of empathy and how to practice a holistic approach." *Besides strictly curriculum based questions, students also reported that this movie made them think about their own life and about death as one of the important events in human life. *"We made a critical review of our life and came to a conclusion that death equates us and that the only thing important at the end of our life is our satisfaction with our own life. At the time of death, we should have no regrets about our life." "This film represents an inspiration for us as human beings - it enables us to be a better human being."*

## Discussion

Using cinemeducation in teaching professionalism proved relevant and useful in our study. Students have recognised the following medico-professional and ethical dimensions of the movie: importance of doctor patient communication, empathy as a mile stone of doctor patient relationship, doctors' selfish personal interests and importance of palliative care. It also made them reflect on their attitudes towards life, death and dying.

Communication is an essential prerequisite when assessing professional behaviour. Although it is not directly listed among professional competencies [18], it is a rather genuine ability of medical professionals. One of its features - respect for others - cannot be achieved without proper communication training and cinemaeducation can be one of the important triggers to learn communication skills. Using verbal and non-verbal communication skills, doctors can effectively express that they care for their patients and that they listen attentively to their concerns. The importance of proper communication being a part of professional behaviour has been recognised also in other teaching programs that dealt with cinemeducation, directly [6,8] or indirectly [5,13].

Students also recognised another important dimension of professionalism - empathy, which is a necessary condition if a doctor wants to develop attributes like honour, integrity and respect for others. This has been showed also in other studies [5,19]. Providing featured roles in a movie like Wit provokes strong natural emotions in students, which are one of the most powerful learning experiences. They should in this case leave our students with positive messages about how to behave professionally as this teaching process gave them an opportunity to learn by the mistakes made by others [7,9].

When it comes to doctors' personal interests, altruism is an important part of professionalism [20]. But it is very hard to teach as it is usually connected to changing the students' values, not just their behaviour, which can provoke their resistance because the line between values and personal attributes is very tiny [3]. By watching a featured movie on doctors' behaviour in emotionally tense situations, students, who are as spectators not at the core of patient management and safe from medico-technical aspects of care, can gain better insights into wrong values and behaviours through the eyes of the patients, which could make them elaborate or even change their professional values. Similar approach to changing values has been demonstrated in other studies [9,11,12]; some of them also used direct and narrow questions to provoke these changes [21].

Palliative care is an emerging medical field where positivism of modern medical technologies loses its power edge and is therefore filled with ethical dilemmas, communication problems and challenges to professional behaviour [22]. In the movie Wit, students recognised many issues from palliative medicine and started to gain understanding of their importance. Besides exposure to difficult ethical dilemmas in a safe environment, this teaching process gave them the opportunity to build their own capacity for managing difficult cases, breaking bad news and facing dying patients with the help and supervision of an experienced teacher to guide them when necessary. A very valuable result of this course is a fact that it has led to students' thinking about their attitudes towards their own life and death. This enables them to grow into reflective doctors [23]. As recognised also in other studies, cinema and theatre can provide social, anthropological, and cultural knowledge about people and in this way help to understand human life [10,24,25]. This, again, can help students to change their wrong values.

The reported dimensions correspond to the main components of medical professionalism, which are excellence, humanism, accountability and altruism [26]. These categories are very broad and may include many different aspects - as seen in this study. The students grasped the core message from the movie and it enhanced their thinking about their future behaviours and values when practising medicine. With such interactive teaching methods, students could become aware that professionalism is more than merely adhering to professional guidelines and possessing an excellent medical knowledge. It is about establishing proper empathetic communication with patients and recognising the role of palliative care which ensures humanism and altruism, about putting aside doctors' personal interests and defining appropriate moral and ethical values and beliefs of doctors which ensures accountability and lead to excellence, an essential part of professionalism [26].

This study has several limitations. The first one is a small sample, which limits us to generalise conclusions to all students. Nevertheless, this was a small study using qualitative methodology which allowed us to explore students' feelings, attitudes and values and as such we can regard it as a representative one for further planning of the curriculum. Another limitation is the fact that results of qualitative analysis were not validated with the students. This is a possible source of misunderstanding between evaluator and students. With this study, the authors did not explore how well the students' knowledge, gained by this elective course, will translate into their later professional life. This should also be assessed by further studies.

## Conclusions

The controlled environment of movies successfully enables students to explore their values, beliefs, and attitudes towards features of professionalism without feeling that their personal integrity had been threatened. Interactive teaching methods could become an indispensible aid in teaching professionalism to new generations.

Further studies about the impact of movies should be done on much larger samples of students and with the use of quantitative methodology.

## Competing interests

The authors declare that they have no competing interests.

## Authors' contributions

ZKK planned the study, performed qualitative analysis and wrote the first and the final draft of the manuscript. JK helped in the designing of the study and coordination and helped to draft the manuscript. All authors read and approved the final version of the manuscript.

## Pre-publication history

The pre-publication history for this paper can be accessed here:

http://www.biomedcentral.com/1472-6920/11/60/prepub
